# Visual evoked potentials waveform analysis to measure intracortical damage in a preclinical model of multiple sclerosis

**DOI:** 10.3389/fncel.2023.1186110

**Published:** 2023-05-31

**Authors:** Silvia Marenna, Elena Rossi, Su-Chun Huang, Valerio Castoldi, Giancarlo Comi, Letizia Leocani

**Affiliations:** ^1^Experimental Neurophysiology Unit, Institute of Experimental Neurology (INSPE)–IRCCS-Scientific Institute San Raffaele, Milan, Italy; ^2^Faculty of Medicine, Università Vita-Salute San Raffaele, Milan, Italy; ^3^Department of Neurorehabilitation Sciences, Casa di Cura Igea, Milan, Italy

**Keywords:** neurophysiology, non-invasive visual evoked potentials, multiple sclerosis, experimental autoimmune encephalomyelitis, intracortical connection

## Abstract

**Introduction:**

Visual evoked potentials (VEPs) are a non-invasive technique routinely used in clinical and preclinical practice. Discussion about inclusion of VEPs in McDonald criteria, used for Multiple Sclerosis (MS) diagnosis, increased the importance of VEP in MS preclinical models. While the interpretation of the N1 peak is recognized, less is known about the first and second positive VEP peaks, P1 and P2, and the implicit time of the different segments. Our hypothesis is that P2 latency delay describes intracortical neurophysiological dysfunction from the visual cortex to the other cortical areas.

**Methods:**

In this work, we analyzed VEP traces that were included in our two recently published papers on Experimental Autoimmune Encephalomyelitis (EAE) mouse model. Compared with these previous publications other VEP peaks, P1 and P2, and the implicit time of components P1-N1, N1-P2 and P1-P2, were analyzed in blind.

**Results:**

Latencies of P2, P1-P2, P1-N1 and N1-P2 were increased in all EAE mice, including group without N1 latency change delay at early time points. In particular, at 7 dpi the P2 latency delay change was significantly higher compared with N1 latency change delay. Moreover, new analysis of these VEP components under the influence of neurostimulation revealed a decrease in P2 delay in stimulated animals.

**Discussion:**

P2 latency delay, P1-P2, P1-N1, and N1-P2 latency changes which reflect intracortical dysfunction, were consistently detected across all EAE groups before N1 change. Results underline the importance of analyzing all VEP components for a complete overview of the neurophysiological visual pathway dysfunction and treatment efficacy.

## 1. Introduction

The visual system is an important region of disease activity in Multiple Sclerosis (MS; Daqqaq, 2021). Inflammation of the optic nerve, in the form of optic neuritis (ON), occurs as the first symptom in 20 to 40% of patients with MS, and about 50 to 70% of patients have an episode of ON within subsequent relapses (Balcer et al., 2014; Miletic-Drakulic et al., 2022). To quantify visual pathway dysfunction, Visual Evoked Potentials (VEPs) are routinely used both in the clinic and in preclinical research. VEPs initiated by strobe flash were noticed in the early years of clinical encephalography in the 1930s (Creel, 2012) and their involvement in clinical practice is continuously growing. VEPs allow to quantify the functional integrity of the visual system from the retina via the optic nerves, optic tracts, to the thalamus, and form projections to the visual cortices (Creel, 2019, Chapter 34, visual evoked potential). Although VEPs usefulness in clinical practice to investigate the visual system is well established, this exam has not yet been included in the Diagnostic Criteria of MS. Therefore, continuous development and characterization of VEP recording in preclinical models of MS are necessary to validate this electrophysiological tool.

For these reasons, over the last few years, our laboratory developed a method to record VEP in rodents with non-invasive electrodes. Thanks to this tool, the visual system in Experimental Autoimmune Encephalomyelitis (EAE) model of MS was investigated in rats and mice (Castoldi et al., 2020; Marenna et al., 2020). In mice, as well as in humans, VEP recording allows to obtain a digital waveform, composed of several peaks, where the attention is usually placed on the N1 peak. The principal measure of the N1 component is the latency of the peak from the start of the stimulus delivery, which reflects the velocity of the signal along the visual pathway until the primary visual cortex (V1). Differently, the N1-P2 amplitude seems to relate to axonal degeneration as well as cortical excitability (You et al., 2011). However, as already mentioned, the VEP waveform is composed of additional positive peaks, namely, P1 and P2. These are usually less considered because of their higher variability. Nonetheless, beyond previous analyses (Marenna et al., 2019), P1 and P2 are more carefully investigated in this work. In particular, our hypothesis is that in mice, as well as in humans, the second positive peak P2, refers to the intracortical connection reflecting possible dysfunctions arising from the visual cortex to other cortical areas (Youssofzadeh et al., 2015). Cortical degeneration is already described in patients (Calabrese et al., 2007) as well as in the EAE model (Hamilton et al., 2019). Analysis of these additional peaks may help not only to characterize cortical degeneration over time, but also could indicate if anterograde or retrograde MS degeneration may be involved (Gabilondo et al., 2014). In addition, as researchers in the field of MS, our focus is not only to develop tools for MS/EAE characterization but also to develop treatments to improve the quality of life on MS patients.

At the moment no cure exists for MS, but multiple agents are FDA-approved to manage the disease. Current therapies can be divided into three groups: treatment for exacerbations, disease-modifying therapies (DMTs), and symptomatic therapies (Gohil, 2015). For example, DMTs that target inflammatory immunopathology can slow the development of functional disabilities but they fail to relieve symptoms. Accordingly, it is most important to develop effective and alternative treatment approaches (Hsu et al., 2021). In particular, over the last years, transcranial direct current stimulation (tDCS) is growing as a possible form of non-pharmacological intervention. TDCS delivers low-current intensity via electrodes on the scalp and modulates the resting membrane potential, increasing or decreasing neuronal firing rates. The delivered current can be positive or negative (anodal or cathodal stimulation, respectively), with opposing effects: anodal tDCS typically increases excitatory post-synaptic potentials that depolarize the neuronal membrane, thus modulating cortical excitability. While cathodal tDCS hyperpolarizes the membrane and induces inhibition (Hiew et al., 2022). It has already been demonstrated that this electrical stimulation carries beneficial effects for patients with major depressive disorder (Bennabi and Haffen, 2018), Alzheimer’s disease (Penolazzi et al., 2015), and stroke (O’Shea et al., 2014), but only a few papers describe improvements in motor-related function or fatigue in MS (Hiew et al., 2022). For this reason, our laboratory investigates tDCS effects in healthy mice and in preclinical MS models. As already published, after a single session, tDCS was able to influence VEP amplitude in wild-type mice (Cambiaghi et al., 2011). Following, our laboratory moved the attention to tDCS applied in EAE mouse model. Indeed, in Marenna et al. (2022), we published a study in which cathodal stimulation prevented VEP delays and optic nerve myelin damage associated with a lower density of inflammatory cells, suggesting an anti-inflammatory effect with potential therapeutic application to be further explored in autoimmune demyelinating diseases. We hypothesize that both types of and their specific parameters, in terms of excitability/inhibitory modulation, can be exploited to decrease or prevent degeneration in the central nervous system.

Overall, the aim of this study is to investigate different VEP components to have a neurophysiological overview of the complete visual pathway in EAE, from the retina to the associated visual cortex. In particular, the possible detection and monitoring of degeneration up to cellular cortical connections could be crucial to refine new treatments.

## 2. Materials and methods

### 2.1. Animals

Traces recorded from female C57BL/6 mice (*n* = 82) aged 6–8 weeks were included in this retrospective data analysis. Two different studies already published were considered. In the first publication (Marenna et al., 2020), to monitor visual impairments, 8 mice were left untouched and considered Healthy controls (H), while 23 mice were immunized and followed up to 37 days post-immunization (dpi). Considering VEP delay, EAE eyes were divided in: EAE eyes With Latency Delay (EAE W LD) and EAE eyes Without Latency Delay (EAE W/O LD). In the second publication (Marenna et al., 2022), to investigate disease treatment modulation, 8 mice were left untouched and considered Healthy controls (H), while 51 mice were immunized (EAE). Here, EAE mice were divided into 16 EAE-Sham, as a control of the active stimulation, 16 EAE-Anodal and 14 EAE-Cathodal. During the experiment, 5 mice died due to the disease severity: 2 mice in the Anodal group and 3 in the Cathodal group. Mice were housed under a controlled 12 h/12 h light/dark cycle, with free access to chow pellets and tap water. These studies were conducted in accordance with the European Community guidelines (Directive 2010/63/EU) and approved by the San Raffaele Institutional Animal Care and Use Committee (IACUC).

### 2.2. Visual evoked potentials recording

Traces analyzed for this work were recorded as previously described. Non-invasive epidermal VEPs were recorded using a 6 mm Ø Ag/AgCl cup electrode placed on the shaveds calp over V1, contralateral to the stimulated eye and a needle electrode was inserted in the nose for reference. The cup was fixed with electro-conductive adhesive paste over one hemisphere, and once completed the recording with the first eye, it was moved to the other hemisphere (Marenna et al., 2019). Mice were anesthetized intraperitoneally (80 mg/kg ketamine, 10 mg/kg xylazine) and adequate level of anesthesia was verified by checking for the presence of tail-pinching reflex. Body temperature was maintained at 36.5 ± 0.5°C by a homoeothermic blanket system with a rectal probe. To dilate the eyes 0.5% tropicamide was used, while ophthalmic gel was applied to prevent drying. Before the procedure, each mouse was placed in a dark room and allowed to adapt to darkness for 5 min. Then, flash stimuli (260 mJ intensity, 10 μs duration, 1 Hz frequency), were delivered with a flash photo stimulator placed 15 cm from the stimulated eye. The non-stimulated eye was covered with a black silicon band. For each session, 3 averages of 20 EEG segments of 500 ms duration starting at the onset of each flash were recorded (Micromed System Plus Evolution, Mogliano Veneto, Italy; sampling frequency 4,096 Hz, bandpass-filter 0.16–1,024, 16 bits coding, bandpass-filter 5–100 Hz, notch filter 50 Hz).

### 2.3. Visual evoked potentials analysis

**FIGURE 1:**
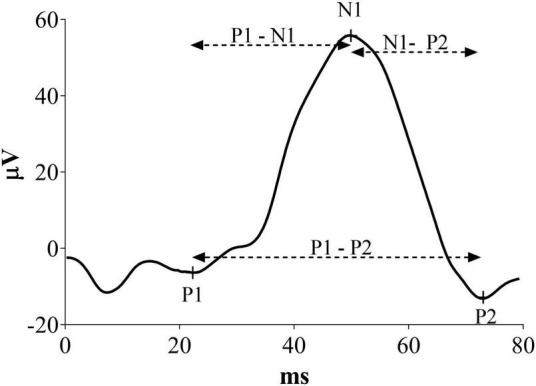
Representative VEP waveform with a description of the peaks indicates the measures considered in this new analysis.

Visual evoked potentials were measured offline, marking latency of P1, N1 and P2 components as illustrated in Figure 1. In this new analysis, P1 was defined as the first positive peak, N1 as the first negative peak and P2 as the second positive peak before the complete signal hyperpolarization. Moreover, P1-N1 and N1-P2 latencies were defined as the time of the second peak minus the first peak. P1-P2 latency was considered as the difference between the two positive peaks. Results in milliseconds were analyzed and converted in percentage change from the baseline.

### 2.4. Transcranial direct current stimulation protocol

The stimulation protocol applied in a previous publication (Marenna et al., 2022) was briefly described as followed. EAE mice were randomized to receive 5 days of tDCS with either cathodal or anodal polarity (from 3 to 7 dpi); the third group of mice was not stimulated and kept as treatment control (EAE-Sham group). TDCS was applied with a current density of 54.4 uA/mm^2^, to awake and freely moving mice for 10 min using a battery-driven, constant current stimulator (BrainSTIM, EMS, Italy). The current intensity was ramped up and down for 10 s instead of switching it on and off directly to avoid a stimulation break effect. Control animals received sham stimulation in which no current was applied but the animal underwent the same manipulations as in the stimulation condition.

### 2.5. Statistical analysis

Statistical analysis was performed with Graphpad Prism (Version 9). Normality data distribution was checked with D’Agostino and Pearson test. When normality data distribution was not assumed, Mix-Model or Kruskal–Wallis test was used followed by *post-hoc* Dunn’s test. Student’s *T*-test was applied to compare N1 and P2 peaks. Data were expressed as mean ± standard error of the mean (SEM). In all tests, a value of *p* < 0.05 was considered significant.

## 3. Results

### 3.1. Positive VEP peaks characterized during disease course

**FIGURE 2:**
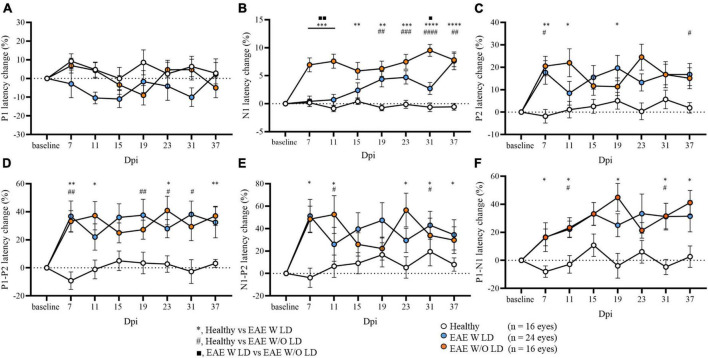
Visual evoked potential (VEP) peaks analysis in EAE until 37 dpi. **(A–F)** VEP components expressed in latency change (%) from the baseline. White dots represent Healthy (*n* = 16 eyes), orange dots represent EAE WLD (*n* = 24 eyes), blue dots represent EAE W/O LD (*n* = 16 eyes). Asterisks *represent significant differences between healthy and EAE W LD; hashes ^#^represent significant differences between Healthy and EAE W/O LD; squares ■, represent significant differences between EAE W LD and EAE W/O LD. Error bars represent the SEM. **(A–F)** Statistics, Mix-model ANOVA followed by Dunn’s *post-hoc* test (*^#■^*p* < 0.05; **^##■■^*p* < 0.01; ***^###^*p* < 0.001; ****^####^*p* < 0.0001).

As published, N1 latency can be used to characterize and stratify EAE mice in the early phase of the disease. In some EAE mice, N1 latency delay was already found at 7 dpi (EAE W LD), while in other mice the delay appeared after the motor onset (EAE W/O LD; Marenna et al., 2020). Maintaining this subdivision, several significant differences were detected in all VEP components analyzed until 37 dpi (Figure 2; Supplementary Table 1). Despite this stratification criterion, P1 latency change did not show significant differences between groups (Figure 2A). On the other hand, P2 latency at 7 dpi, change was significantly increased in both EAE groups compared to Healthy mice but not among EAE groups (Figure 2C; Healthy vs. EAE W LD, *p* = 0.0016; Healthy vs. EAE W/O LD, *p* = 0.0240; EAE W LD vs. EAE W/O LD, *p* = 0.920). Moreover, at 7 dpi, P1-P2 and N1-P2 latency change (%) in both EAE groups were significantly different compared with Healthy (Figure 2D; P1-P2: Healthy vs. EAE W LS, *p* = 0.0023; Healthy vs. EAE W/O LD, *p* = 0.0086. Figure 2E; N1-P2: Healthy vs. EAE W LD, *p* = 0.0041; Healthy vs. EAE W/O LD, *p* = 0.0229).

**FIGURE 3:**
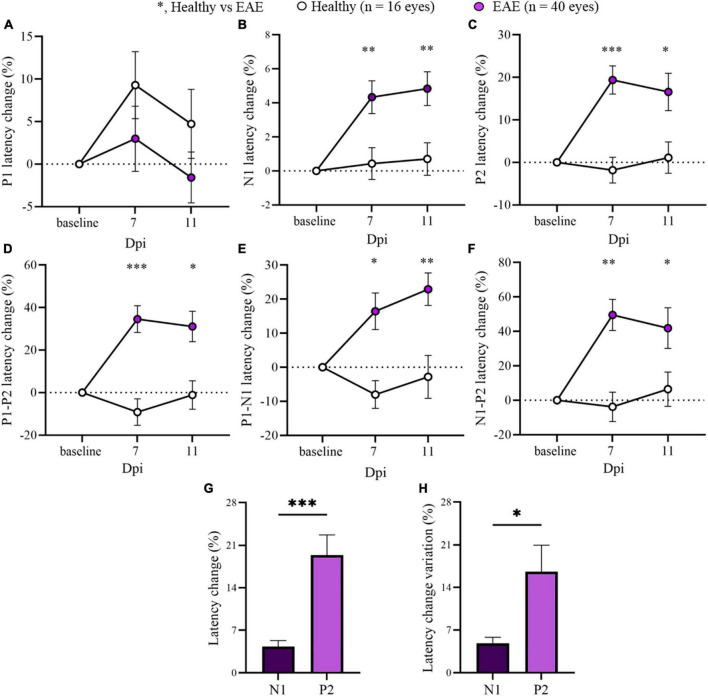
Visual evoked potentials (VEP) peaks analysis in pooled EAE mice at baseline, 7 and 11 dpi. **(A–F)** VEP components expressed in latency change (%) from the baseline. White dots represent Healthy (*n* = 16 eyes), violet dots represent pooled EAE (*n* = 40 eyes). Error bars represent the SEM. **(A–F)** statistics, Mix-model ANOVA followed by Dunn’s *post-hoc* test. **(G,H)** Statistics, Student *T*-test (*p < 0.05; **p < 0.01; ***p < 0.001).

Considering the stability of P1 and P2 latencies at early time points, the two EAE groups were pooled to focus the attention on 7 and 11 dpi for the overall EAE group. A significant delay in the latency change (%) was detected in all EAE peaks compared with Healthy (Figure 3; Supplementary Table 2). Moreover, P2 latency change variation was significantly increased in contrast to N1 (*p* = 0.0106; Figure 3H).

### 3.2. Positive VEP peaks modulated by tDCS

**FIGURE 4:**
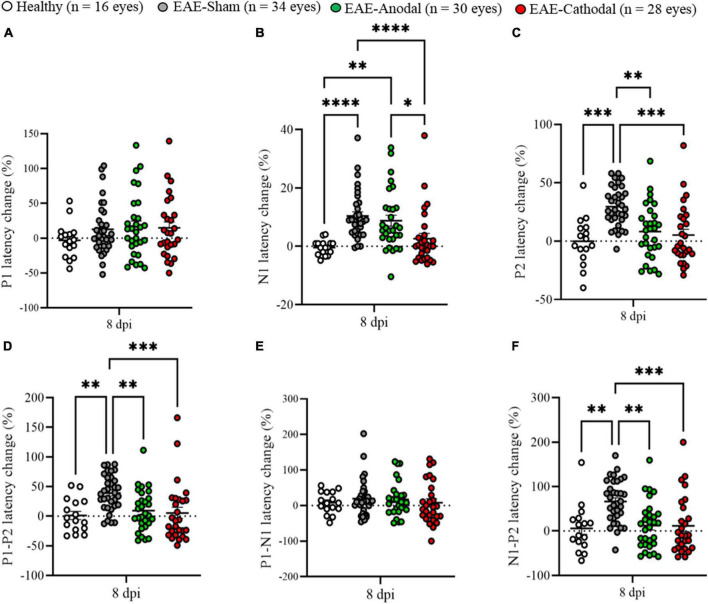
Visual evoked potentials (VEP) peak analysis in EAE groups under tDCS treatment. **(A–F)** VEP components expressed in percentage of latency change from baseline. White dots represent Healthy (*n* = 16 eyes), gray dots represent EAE-Sham (*n* = 34 eyes), green dots represent EAE-Anodal (*n* = 30 eyes), red dots represent EAE-Cathodal (*n* = 28 eyes). Error bars represent the SEM. **(A–F)** Statistics, Kruskal–Wallis test followed by Dunn’s *post-hoc* test (*p < 0.05; **p < 0.01; ***p < 0.001; ****p < 0.0001).

To evaluate the possible effects of tDCS on positive VEP peaks, Kruskal–Wallis test on latency change at 8 dpi was performed (Figure 4). Concerning P1 latency change, no significant differences were detected in mice that received active stimulation (Supplementary Table 3). As already demonstrated, N1 latency change delay was significantly decreased in EAE-Cathodal mice compared with EAE-Sham and EAE-Anodal (Figure 4B; Marenna et al., 2022). However, P2 latency change was significantly reduced in EAE-Anodal and EAE–Cathodal mice compared with EAE-Sham (Figure 4C; EAE-Sham vs. EAE-Anodal, *p* = 0.0013; EAE-Sham vs. EAE-Cathodal, *p* = 0.0001) and no significant differences were found in EAE-treated mice compared with Healthy (Healthy vs. EAE-Anodal, *p* > 0.9999; Healthy vs. EAE-Cathodal, *p* > 0.9999; Supplementary Table 3). In addition, P1-P2 and N1-P2 latency change showed a significant decrease in EAE-Anodal and EAE-Cathodal mice compared with EAE-Sham (Figure 4D; P1-P2, EAE-Sham vs. EAE-Anodal, *p* = 0.0059; EAE-Sham vs. EAE-Cathodal, *p* = 0.0002. Figure 4F; N1-P2, EAE-Sham vs. EAE-Anodal, *p* = 0.0011; EAE-Sham vs. EAE-Cathodal, *p* = 0.0004).

## 4. Discussion

A large body of literature nowadays confirms that VEPs represent a remarkable non-invasive electrophysiological tool able to characterize visual pathway dysfunction longitudinally. As already published in humans as well as in mice, the visual cortex is composed of a primary area, V1, with other accessory areas in the cortex (Palagina et al., 2017; Huff et al., 2022). Several neuroimaging studies have investigated the spatiotemporal characteristics of neural generators in the visual system under a checkerboard pattern reversal process (Barnikol et al., 2006; Di Russo et al., 2007). For instance, using a combined VEP-fMRI source analysis, Di Russo et al. (2007) showed that, in humans, early pattern reversal VEP components (N75 and P100) are generated in the medial occipital cortex (V1) while the N150 component is generated by contribution from several areas, particularly the parietal lobe [motion selective (MT/V5) area] as the major contributor, while the mid-occipital (V3A) and ventral occipital (V4/V8) cortices as minor contributors (Youssofzadeh et al., 2015). On the other hand, only a few studies in mice described the feed forward sensory signal from V1 (Jia et al., 2021). Similarities between human and mouse visual systems, in the hierarchy and signal recording, allowed us to consider the clinical tools for visual monitoring on mice and vice-versa, effectively improving the understanding of the visual system in preclinical health and disease, with translational value back to the clinic. In fact, in our laboratory VEP recording takes advantage of small pediatric epidermal electrodes adapted for rodents (Santangelo et al., 2018; Marenna et al., 2019). As in humans, the outcome of these recordings is a specific wave with several peaks. The central peak N1 (P100 in the human) can be considered a biomarker of visual function (Marenna et al., 2019) from the eye up to the primary visual cortex. In MS patients P100 delay can detect optic neuritis in the early phase or during the disease (Leocani et al., 2018). At the same time, N1 peak in rodents reflects demyelination, axonal loss, and cortical excitability changes in preclinical MS models, such as the EAE (Marenna et al., 2019; Castoldi et al., 2020). Due to its signal stability, several research groups consider in their studies the principal VEP component N1, however, in this work we focused the attention on P1 and P2 peaks. It is well-established that electrophysiological recordings can reflect intracortical connections (Aggarwal et al., 2022; Claar et al., 2022; Jia et al., 2022) and indeed LeBlanc et al. (2015) reported that VEP latency was related to specific cortical alterations.

Performing a blind retrospective analysis on our VEPs recorded in EAE mice in both studies of EAE visual characterization and tDCS treatment efficacy, P1 latency change from its baseline showed no significant differences between groups despite the alteration of N1 and P2 components. It is already known that changing the visual light contrast time, P1 latency speed depends on the type of axons that are activated in the visual cortex (Oka et al., 2011). P1 is the first peak of the VEP waveform, representing the first electrical signal recorded in the visual cortex. In the central nervous system, axons with larger diameter conduct at higher speeds (Suminaite et al., 2019) and P1 could reflect these larger axons in the visual system. Accordingly, the lack of P1 alterations may indicate that the visual system of EAE mice was not completely compromised and intact large axons were still intact and able to transmit the signal efficiently. On the contrary, smaller and demyelinated axons showed a reduced velocity, together with an increase in P1-N1 implicit time.

Switching the discussion to the P2 component and taking into account the EAE stratification as already described (Marenna et al., 2020), the latency change was increased in both EAE W LD and EAE W/O LD groups compared with Healthy. This delay was unexpected, especially for the EAE group for which N1 latency change was not significantly increased at early time points (EAE W/O LD), pointing to normal conduction and preserved visual system at that stage. Considering this interesting finding, it could be speculated that the presence of cortical alterations deriving from cellular dysfunction of intracortical areas may drive an inside-out like pathology. Therefore, the visual system may be impaired not only at peripheral sites, but also in higher cortical areas, which would be compromised due to the ongoing disease within other areas of the brain. Even in this case, cortical degeneration in EAE is often detected by histology (Girolamo et al., 2011), MRI (Alomair et al., 2021), PET (Guglielmetti et al., 2022), or both imaging methods (Belolli et al., 2018) during disease. This important feature is under investigation at the beginning of MS clinical studies because cortical lesions contribute to clinical symptoms and disease progression in chronic MS (Kutzelnigg et al., 2005). However, the pathogenesis of cortical lesions is largely unknown and the difference between cortical and white matter lesions seems to be a crucial point in patients (Markler et al., 2006). Coming back to our study, the novel idea consists in applying non-invasive VEPs to investigate not only the transmission through the proximal visual pathway but also the intracortical visual dysfunction in the early stages of optic neuritis. Currently, some studies describe cortical structural degeneration from 14 to 40 dpi (Alomair et al., 2021; Guglielmetti et al., 2022), but our data raise the possibility to detect cortical dysfunction in the EAE model as early as 7 dpi.

Another important point to consider deals with the results obtained after the blinded analysis of VEP waveforms recorded after 5 days of tDCS treatment, with particular attention to P2 latency change recorded in the EAE-Anodal group. Through the analysis of N1 latency change from its baseline (Marenna et al., 2022), the EAE-Anodal group showed no amelioration of this component after tDCS treatment. However, the investigation of P2, P1-P2, and N1-P2 latencies revealed a significant difference in EAE-Anodal and EAE-Cathodal groups compared to EAE-Sham, and no differences compared with Healthy controls. These data demonstrate that despite N1 latency in EAE-Anodal mice was not significantly reduced, intracortical connections were modulated by anodal stimulation, which improved the neuronal activity toward physiological levels. Moreover, the reduction of implicit time of the peak could be due to the increased cellular excitability induced by Anodal stimulation in the brain. Polarity-dependent effects of tDCS are complex. On one hand, as already published (Marenna et al., 2020), beneficial results by cathodal tDCS were obtained, where the stimulation was able to reduce microglia/macrophage cells in the optic nerve and probably also in the brain. Thus, by reducing the inflammatory state, both N1 and P2 latencies from EAE mice where modulated to similar levels as the Healthy group compared with the EAE-Sham group. Association of tDCS with reduced inflammation is also reported a model of middle cerebral artery occlusion (MCAO) where cathodal stimulation inhibited the activation of astrocyte and microglia cells, as well as neuroinflammation and apoptosis (Zhang et al., 2020). Further, contrarily to high-charge, low-charge density anodal tDCS was also found to down-regulate microglia in a multisession tDCS protocol in wild-type mice (Pikhovych et al., 2016), suggesting a highly complex mechanism for electrical stimulation which may depend on multiple parameters in addition to polarity, such as duration, density charge, and localization. The present data analysis may indicate that whereas 5 days of Cathodal tDCS strongly modulated N1, the treatment with Anodal tDCS produced an effect localized in the brain, revealed by the P2 component. Indeed, whilst the amplitude of VEPs was increased (anodal) or decreased (cathodal) after a single session of tDCS (Cambiaghi et al., 2011), amplitude changes where not detected after a 5 days treatment (Marenna et al., 2022), suggesting alternative mechanisms of action of repeated tDCS. Nonetheless, the current findings indicate that tDCS does have restorative properties at functional level, which can be detected by electrophysiology.

Notwithstanding, we are aware that this study presents some limitations. First of all, it is necessary to underline that VEPs were recorded in mice under anesthesia and signals may change in awake mice. Nonetheless, as already published the difference between awake and anesthetized mice is the latency of the peak and not its stability (Tomiyama et al., 2016). Indeed, there is indication that VEPs recorded in freely moving the peak investigated was maintained (Perenboom et al., 2021). Thus, available evidence suggests that application of this analysis to VEPs recorded in awake mice may be possible. Secondly, this is a retrospective work and we were not able to perform histological analysis on mouse brain. We are aware that both analyses and the hypothesis derived from this work would require further histological validation to support the data. For this reason, future experiments will involve examination of brain damage and change of excitability by specific markers such as Myelin Binding Protein (MBP) and Post-synaptic Density Protein 95 (PSD95). Nonetheless, our findings strongly suggest that P2 and N1-P2 implicit time are a highly sensitive signal of intracortical activity in V1 and associated cortices in neurophysiological studies of the visual pathway. In conclusion, our findings indicate the importance of considering and investigate all VEP peaks in preclinical models of MS, paying particular attention to P2 as a biomarker for cortical function. Indeed, the N1-P2 as a measure of intracortical conduction at the early stages of the EAE disease, and may be relevant for detection of visual dysfunctions in other preclinical models with visual pathway involvement.

## Data availability statement

The raw data supporting the conclusions of this article will be made available by the authors, without undue reservation.

## Ethics statement

This animal study was reviewed and approved by the European Community guidelines (Directive 2010/63/EU) and approved by the San Raffaele Institutional Animal Care and Use Committee (IACUC).

## Author contributions

SM and LL contribution to the conception of the theory and manuscript preparation. SM, S-CH, and VC contribution to the experiment. SM and ER prepared the manuscript. GC and LL supervised the project. All authors read and approved the final manuscript.
