# Supplementary material for: Visual evoked potentials waveform analysis to measure intracortical damage in a preclinical model of multiple sclerosis
Source: Front Cell Neurosci. 2023 May 31;17:1186110. doi: 10.3389/fncel.2023.1186110 (PMC10264580; doi:10.3389/fncel.2023.1186110)
Supplement: Supplementary file 1 [file Data_Sheet_1.docx]

**SUPPLEMENTARY**

**TABLE**

Table 1. p values referred to the latency change (%) until 37 dpi.

|  | | Time points | | | | | | | | | | | | | |
| --- | --- | --- | --- | --- | --- | --- | --- | --- | --- | --- | --- | --- | --- | --- | --- |
| **Peak** | **Groups** | **7** | | **11** | | **15** | | **19** | | **23** | | **31** | | **37** | |
| **N1** | Healthy vs. EAE W LD | *** | 0,0009 | *** | 0,0001 | ** | 0,0077 | ** | 0,0042 | *** | 0,0005 | **** | <0,0001 | **** | <0,0001 |
|  | Healthy vs. EAE W/O LD | ns | 0,9792 | ns | 0,366 | ns | 0,3858 | ** | 0,0076 | *** | 0,0006 | * | 0,0365 | ** | 0,0012 |
|  | EAE W LD vs. EAE W/O LD | ** | 0,0034 | ** | 0,0011 | ns | 0,1273 | ns | 0,6866 | ns | 0,3698 | **** | <0,0001 | ns | 0,9976 |
| **P2** | Healthy vs. EAE W LD | ** | 0,0016 | * | 0,0263 | ns | 0,0905 | ns | 0,4703 | * | 0,0111 | ns | 0,342 | ns | 0,0641 |
|  | Healthy vs. EAE W/O LD | * | 0,0236 | ns | 0,534 | ns | 0,1746 | ns | 0,0553 | ns | 0,155 | ns | 0,2171 | * | 0,0407 |
|  | EAE W LD vs. EAE W/O LD | ns | 0,92 | ns | 0,3331 | ns | 0,8298 | ns | 0,5428 | ns | 0,2927 | ns | >0,9999 | ns | 0,9624 |
| **P1-P2** | Healthy vs. EAE W LD | ** | 0,0023 | * | 0,0131 | ns | 0,1337 | ns | 0,0946 | * | 0,0145 | ns | 0,0844 | ** | 0,0021 |
|  | Healthy vs. EAE W/O D | ** | 0,0086 | ns | 0,1437 | ns | 0,1034 | ** | 0,0094 | * | 0,0434 | * | 0,0172 | ns | 0,0563 |
|  | EAE W LD vs. EAE W/O D | ns | 0,9691 | ns | 0,6168 | ns | 0,6779 | ns | 0,7589 | ns | 0,5972 | ns | 0,7842 | ns | 0,9358 |
| **P1-N1** | Healthy vs. EAE W LD | * | 0,0463 | * | 0,0386 | ns | 0,2409 | * | 0,0157 | ns | 0,1324 | * | 0,0428 | * | 0,0156 |
|  | Healthy vs. EAE W/O LD | ns | 0,1214 | * | 0,0367 | ns | 0,1925 | ns | 0,0639 | ns | 0,2789 | * | 0,0131 | ns | 0,163 |
|  | EAE W LD vs. EAE W/O LD | ns | 0,9998 | ns | 0,994 | ns | >0,9999 | ns | 0,4365 | ns | 0,768 | ns | 0,9998 | ns | 0,8337 |
| **N1-P2** | Healthy vs. EAE W LD | ** | 0,0041 | ns | 0,0659 | ns | 0,2648 | ns | 0,915 | * | 0,0259 | ns | 0,7435 | ns | 0,2281 |
|  | Healthy vs. EAE W/O LD | * | 0,0229 | ns | 0,5725 | ns | 0,2954 | ns | 0,1467 | ns | 0,3632 | ns | 0,439 | ns | 0,2303 |
|  | EAE W LD vs. EAE W/O LD | ns | 0,9878 | ns | 0,564 | ns | 0,7225 | ns | 0,4613 | ns | 0,3348 | ns | 0,8579 | ns | 0,9497 |

**Table 2.** p values referred to Healthy vs. pooled EAE mice at 7 and 11 dpi.

|  | **Peak** | **N1** | **P2** | **P1-P2** | **P1-N1** | **N1-P2** |
| --- | --- | --- | --- | --- | --- | --- |
|  | **Groups** | Healthy vs. EAE | Healthy vs. EAE | Healthy vs. EAE | Healthy vs. EAE | Healthy vs. EAE |
| **Time points** | **7** | ** | *** | *** | * | ** |
|  |  | 0,0018 | 0,0007 | 0,0004 | 0,0112 | 0,0017 |
|  | **11** | ** | * | * | ** | * |
|  |  | 0,0015 | 0,0169 | 0,0102 | 0,0072 | 0,0441 |

**Table 3.** p values referred to Healthy vs. EAE mice under tDCS treatment

| **Peaks** | **Group** | **Sign** | **Value** |
| --- | --- | --- | --- |
| **N1** | Healthy vs. EAE-Sham | **** | <0,0001 |
|  | Healthy vs. EAE-Anodal | ** | 0,0018 |
|  | Healthy vs. EAE-Cathodal | ns | >0,9999 |
|  | EAE-Sham vs. EAE-Anodal | ns | >0,9999 |
|  | EAE-Sham vs. EAE-Cathodal | **** | <0,0001 |
|  | EAE-Anodal vs. EAE-Cathodal | * | 0,0115 |
| **P2** | Healthy vs. EAE-Sham | *** | 0,0002 |
|  | Healthy vs. EAE-Anodal | ns | >0,9999 |
|  | Healthy vs. EAE-Cathodal | ns | >0,9999 |
|  | EAE-Sham vs. EAE-Anodal | ** | 0,0013 |
|  | EAE-Sham vs. EAE-Cathodal | *** | 0,0001 |
|  | EAE-Anodal vs. EAE-Cathodal | ns | >0,9999 |
| **P1-P2** | Healthy vs. EAE-Sham | ** | 0,0044 |
|  | Healthy vs. EAE-Anodal | ns | >0,9999 |
|  | Healthy vs. EAE-Cathodal | ns | >0,9999 |
|  | EAE-Sham vs. EAE-Anodal | ** | 0,0059 |
|  | EAE-Sham vs. EAE-Cathodal | *** | 0,0002 |
|  | EAE-Anodal vs. EAE-Cathodal | ns | >0,9999 |
| **N1-P2** | Healthy vs. EAE-Sham | ** | 0,0028 |
|  | Healthy vs. EAE-Anodal | ns | >0,9999 |
|  | Healthy vs. EAE-Cathodal | ns | >0,9999 |
|  | EAE-Sham vs. EAE-Anodal | ** | 0,0011 |
|  | EAE-Sham vs. EAE-Cathodal | *** | 0,0004 |
|  | EAE-Anodal vs. EAE-Cathodal | ns | >0,9999 |
| **P1-N1 vs.N1-P2** | Healthy vs. EAE-Sham | * | 0,041 |
|  | Healthy vs. EAE-Anodal | ns | >0,9999 |
|  | Healthy vs. EAE-Cathodal | ns | >0,9999 |
|  | EAE-Sham vs. EAE-Anodal | * | 0,0171 |
|  | EAE-Sham vs. EAE-Cathodal | * | 0,036 |
|  | EAE-Anodal vs. EAE-Cathodal | ns | >0,9999 |
